# Influence of Mn Element on the Microstructure Evolution and Mechanical Properties of High-Strength Iron-Based Alloy Coatings Fabricated Through Laser Cladding

**DOI:** 10.3390/nano16140860

**Published:** 2026-07-13

**Authors:** Xinghua Wang, Rui Ding, Chenhui Hu, Linlin Qin, Yanming Wu, Liming Liu, Xin Lin

**Affiliations:** 1 School of Materials Science and Engineering, Dalian University of Technology, Dalian 116024, China; wangxh1717@126.com; 2 Luoyang Ship Material Research Institute, China State Shipbuilding Corporation Limited, Luoyang 471023, China; 13307084023@163.com (R.D.);; 3 Innovation Center for Welding Technology of Large Components, Luoyang Ship Material Research Institute, Luoyang 471023, China; 4 School of Material Science and Engineering, Northwestern Polytechnical University, Xi’an 710072, China

**Keywords:** laser cladding, Fe-based alloy coating, Mn content, microstructural evolution, mechanical properties

## Abstract

Manganese can provide strong solid-solution strengthening in austenite, thereby effectively enhancing the strength and toughness of Fe-based coatings. However, the underlying mechanism remains unclear. In this study, building on previously developed high-strength, hard Fe-based alloy coatings produced via laser cladding, the influence of Mn content on the microstructural evolution and mechanical properties is systematically investigated using XRD, SEM, TEM, microhardness testing, and a reciprocating friction and wear testing machine. The results show that the coatings fabricated from three iron-based alloy powders are primarily composed of a martensitic matrix, retained austenite, and various MxC-type eutectic carbides (such as MC, M_2_C, M_6_C). With increasing Mn content, the MC-type carbides gradually change from a blocky morphology to a fishbone-like structure, while the originally fishbone-like M_6_C-type carbides first become blocky and eventually turn into fine granules. Meanwhile, the sizes of all three types of carbides are refined. Especially, after the addition of Mn, dispersed second-phase particles (Mn-Si-O) precipitate in the coatings. The coating with 2.0 wt.% Mn exhibits the best comprehensive mechanical properties: an average microhardness of 925 ± 8 HV_0.5_ and a minimum wear rate of 0.78 × 10^−6^ mm^3^/N·m.

## 1. Introduction

38CrMoAl steel possesses high strength, excellent toughness and hardenability, as well as low cost, and has been widely used in critical fields including aerospace, marine vessels, and precision molds [1]. However, the high temperatures, high pressures, and impact wear encountered in extreme operating environments impose increasingly stringent demands on the fatigue strength, hardness, and wear resistance of component surfaces. The low microhardness (200~300 HV), high and unstable coefficient of friction, and poor wear resistance of 38CrMoAl steel at high temperatures severely limit the service life and reliability of components subjected to high-impact, high-temperature, or severe-wear environments [2,3,4].

The application of surface engineering techniques to fabricate high-performance hardfacing coatings on wear-prone areas of mechanical components represents one of the most cost-effective and technically feasible strategies for improving the properties of the substrate material and extending the overall service life of the component. Laser cladding has emerged as a cutting-edge method for producing such coatings because of its high energy density, precise and controllable heat input, rapid cooling rates, metallurgical bonding to the substrate, and low dilution ratio [5,6,7,8]. During the process, a high-energy laser beam rapidly fuses the coating material onto the surface of a substrate, thereby enhancing the surface functionality or repairing surface damage. Whether a repaired component can meet the performance requirements for returning to service depends on the quality of the laser-deposited coating. Therefore, significantly improving coating performance remains a challenging issue [9].

Currently, research on fabricating high-performance reinforced coatings by laser cladding approaches the problem from multiple perspectives to evaluate the comprehensive properties and overall quality of these coatings. Common strategies include designing the coating material composition [10,11], optimizing process parameters [12,13,14], applying auxiliary energy fields [15], and performing post-cladding heat treatment [16,17] to enhance the comprehensive performance of laser-deposited coatings. Among these, the type of coating material and its elemental composition are the key factors determining the coating performance. Hence, the rational selection of the alloying elements and concentrations is of paramount importance. Several researchers have developed new coating materials while optimizing the existing ones and have systematically evaluated the performance of the resulting laser-clad layers [18,19]. High-toughness iron-based alloy powder, when used as a high-strength coating material, can yield coatings with high strength and hardness. These properties are mainly attributed to the martensitic matrix and the hard reinforcing phases uniformly distributed within it. The alloys are therefore typically Fe-C-X component systems, where X represents a strong carbide-forming element such as chromium, tungsten, niobium or vanadium [20,21]. However, the hard reinforcing phases in such coatings tend to be highly brittle and are prone to forming continuous network carbides at grain boundaries, resulting in insufficient toughness and poor crack resistance [22]. It has been found that introducing boron can effectively modify the morphology of carbides in high-strength iron-based alloy coatings, transforming the grain-boundary network carbides into eutectic carbides and thus significantly improving the overall toughness of the coatings [23,24]. Meanwhile, manganese, as a common alloying element in steel, provides a strong solid-solution strengthening in austenite, thereby effectively enhancing the strength and toughness of the coating [25]. Therefore, it is crucial to investigate how the addition of manganese enhances the properties of Fe-C-B-X multicomponent iron-based alloy coatings [26].

Existing research on high-strength and -hardness iron-based alloy coatings has primarily focused on process parameter optimization and post-cladding heat treatment. However, the influence of manganese on microstructural evolution and mechanical properties of such coatings produced by laser cladding remains unclear. Therefore, comparative experiments are conducted by adding different Mn contents to elucidate the effects of manganese on the microstructure evolution and mechanical properties of Fe-C-B-X multicomponent iron-based alloy coatings fabricated through laser cladding.

## 2. Materials and Methods

### 2.1. Materials

**Figure 1:**
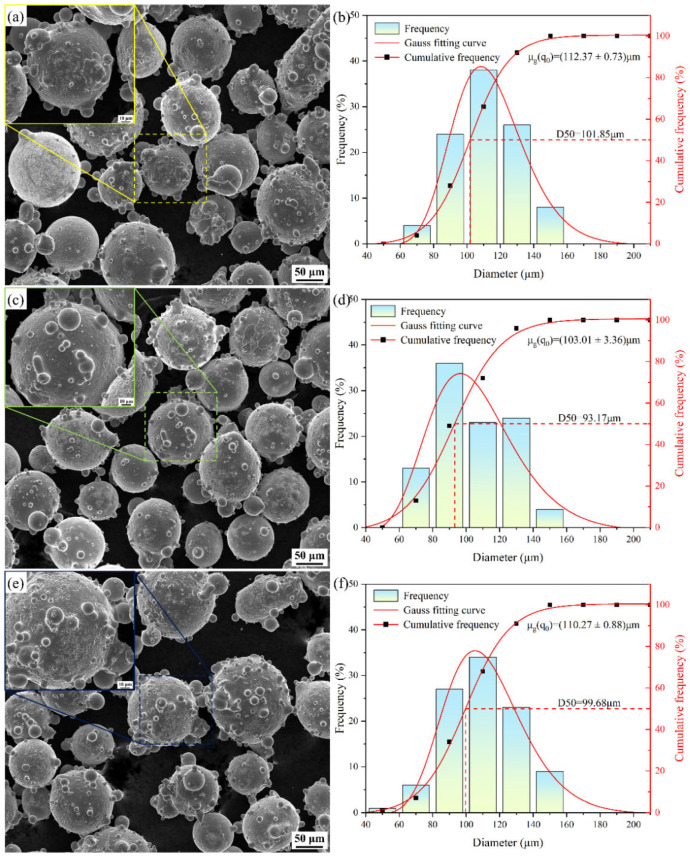
SEM morphology of three Fe-based alloy powders and diameter distribution: (**a**,**b**) 0Mn, (**c**,**d**) 1Mn. (**e**,**f**) 2Mn.

38CrMoAl steel produced by Northeast Special Steel Group Co., Ltd., Dalian, China was selected as the substrate material based on practical engineering requirements. It was machined into test plates with a diameter of 160 mm and a thickness of 20 mm for subsequent coating deposition. Three Fe-C-B-X multi-component iron-based alloy powders with Mn contents of 0.0%, 1.0%, and 2.0% were used as cladding materials, and designated as 0Mn, 1Mn, and 2Mn, respectively. The SEM morphology and particle size distribution of the powders are shown in Figure 1. The particles are approximately spherical, with some fine satellite particles adhering to their surface. The particle sizes range from 53 to 150 μm, with the median particle sizes being 101.85, 93.17, and 99.68 μm, respectively. The main chemical compositions of the substrate and the three alloy powders are listed in Table 1.

**Figure 2:**
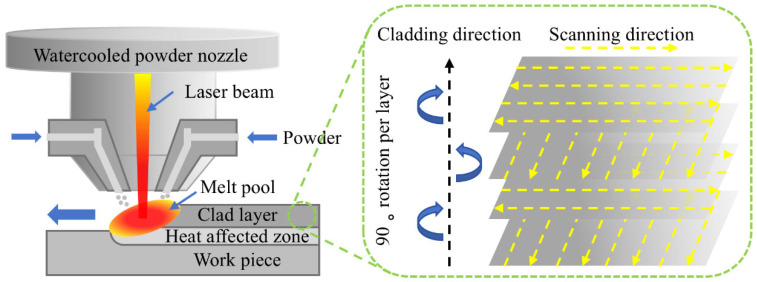
Schematic diagram of laser cladding process.

Coating deposition experiments were conducted using a self-developed, improved four-beam coaxial powder-fed laser solid forming system. The process parameters are detailed in Table 2. A bidirectional weaving scanning strategy was employed for coating fabrication, and the specific scanning path is illustrated in Figure 2 [27]. Using the above process parameters and scan path, six layers were deposited onto the surface of the 38CrMoAl substrate, resulting in a total coating thickness of approximately 3.6 mm. To eliminate variations in the chemical composition and overall properties of the substrate material, all substrates were taken from the same batch of 38CrMoAl steel. This addresses variability from steel production and heat treatment processes.

Before laser cladding, the substrate surface was ground, polished, and wiped with acetone to remove the oxide layer and surface grease, thereby improving the laser absorption. The three alloy powders were dried at 100 °C for 2 h to ensure good flowability during cladding. The substrate was preheated to 200 °C using a ceramic infrared heater before the experiments. To prevent thermal stress concentration and reduce the susceptibility to cracking, a contact thermometer was used to monitor the substrate temperature intermittently during the cladding process, ensuring accurate preheating temperature and interlayer temperature control. High-purity argon (99.99% purity) was used as the shielding gas at a flow rate of 15 L/min to protect the melt pool from oxidation.

### 2.2. Characterization Methods

In this study, all microstructural sections of the coated specimens were taken as cross-sections perpendicular to the coating thickness. For microstructural observation, the specimens were sequentially ground with SiC abrasive papers under water, polished using diamond paste, and then chemically etched with aqua regia. The microstructures were examined with an FEI Scios2 dual-beam scanning electron microscope (SEM) (FEI Company, Hillsboro, OR, USA) equipped with an energy-dispersive spectrometer (EDS). Crystal structure and major phase constituents were analyzed using a BRUKER D8 ADVANCE X-ray diffractometer (XRD) (Bruker AXS GmbH, Karlsruhe, Baden-Württemberg, Germany) with Cu kα radiation (operated at 40 kV and 40 mA), a LynxEye solid-state detector, a scanning speed of 1°/min, and a 2θ angle range of 30 to 100° to identify phase composition. A Talos F200X transmission electron microscope (TEM) (Thermo Fisher Scientific, Brno, South Moravian Region, Czech Republic) was employed to observe the microstructure and crystal structure of the coatings with different Mn contents. To prepare TEM specimens, a region of interest was first identified by SEM, and foils were extracted using a focused ion beam (FIB). A protective Pt layer was deposited over the selected area to avoid ion beam damage. High-energy gallium ion beams were then used for rough milling and lift-out of the target region. The extracted sample was transferred to a TEM grid and further thinned using a low-current ion beam until electron transparency (thickness < 100 nm).

### 2.3. Mechanical Property Testing

Micro-Vickers hardness measurements were carried out using a Wilson VH3300 hardness tester under a load of 4.9 N and a dwell time of 10 s. Two sets of measurements were performed: (i) a hardness depth profile along the coating cross-section, starting at 0.3 mm from the coating surface with a spacing of 0.3 mm between indentations, covering a total of 15 points; (ii) in-plane hardness on a plane located 0.6 mm below the coating surface. To improve accuracy, five indentations spaced 0.6 mm apart were made on this plane; after discarding the two most divergent values, the average of the remaining three was taken as the result.

**Figure 3:**
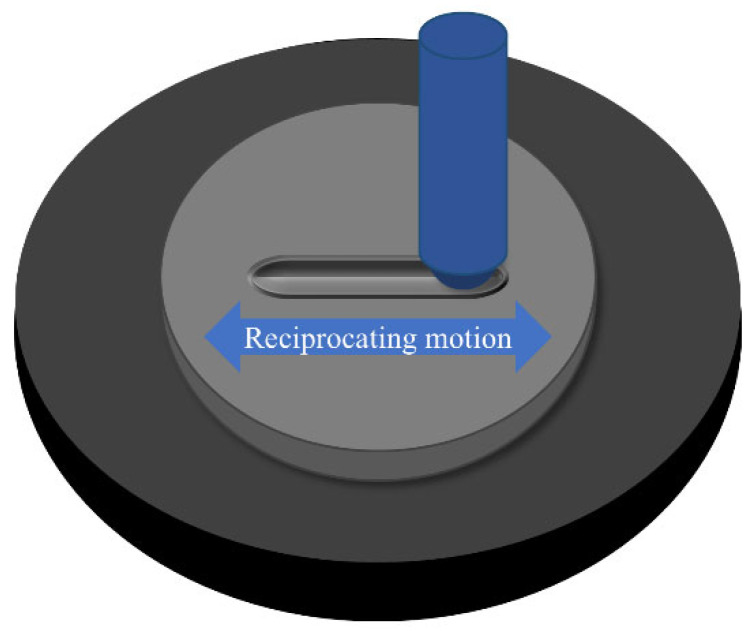
Schematic diagram of friction wear test.

Reciprocating friction and wear tests were conducted on specimens cut from the coatings (φ20 mm × 3 mm) using a reciprocating tribometer. Tests were performed at ambient temperature under a load of 6 N for 30 min. Si_3_N_4_ ceramic balls with a diameter of 4 mm were chosen as the counterpart because their high elastic modulus minimizes deformation and ensures consistent contact with the coating [28]. The friction head was performed in a reciprocating motion at a frequency of 6 Hz and a stroke length of 5 mm. To minimize experimental scatter, three replicate tests were performed for each coating. The wear test configuration is illustrated in Figure 3. After testing, the 2D and 3D wear scar profiles were examined using a laser scanning confocal microscope. The wear rate was calculated by the following equation:

(1) $$\begin{array}{c} W_{R}=\frac{V}{F\cdot f\cdot t\cdot l} \end{array}$$

where *W_R_* is the wear rate (mm^3^/N·m), *V* is the wear volume (mm^3^), *F* is the load (N), *f* is the reciprocating frequency (Hz), *t* is the loading time (s), and *l* is the single stroke (mm).

## 3. Results and Discussions

### 3.1. Microstructure Characterization

#### 3.1.1. Phase Identification

**Figure 4:**
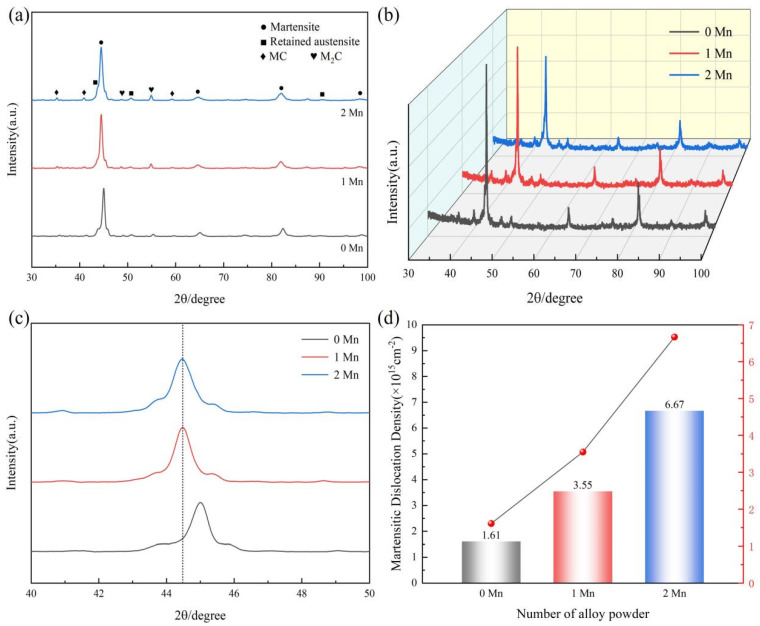
X-ray diffraction patterns (**a**) and 3D waterfall plots (**b**) of iron-based alloy coatings with different Mn contents, and a partial enlarged view in (**a**) from 40° to 50° diffraction angle (**c**), and the trend in martensite dislocation density for the three coatings (**d**).

Figure 4 presents the XRD patterns and 3D waterfall plots of the coatings fabricated from the three alloy powders with different Mn contents. As shown in Figure 4a,b, the phase constituents of the coatings with Mn mass fractions of 0, 1.0, and 2.0 wt.% are generally similar. All consist of a martensitic matrix, retained austenite, and MC-type carbides and M_2_C-type carbides.

With the addition of Mn, the main diffraction peak of the coating shifts markedly toward smaller angles, as shown in Figure 4c. This shift occurs because the atomic radius of Mn is larger than that of the matrix elements. When Mn atoms dissolve into the matrix substitutionally or interstitially, they alter the interatomic forces, causing localized lattice distortion and stress, and thereby increasing both the lattice constant and the interplanar spacing. Furthermore, as an austenite-stabilizing element, Mn lowers the martensite transformation temperature. During the laser cladding process, this promotes the formation of martensite with a higher Mn content, which typically possesses a larger lattice constant, further contributing to the leftward shift of the main peak [29]. Figure 4d shows the variation in martensitic dislocation density in the coatings with different Mn contents. The dislocation density increases continuously with increasing Mn content, reaching a maximum value of 6.67 × 10^15^ cm^−2^ at a Mn mass fraction of 2.0 wt.%. At the same time, the rapid solidification inherent to laser cladding suppresses dislocation recovery, and Mn segregation may generate additional local stresses. Together, these effects result in a pronounced increase in the martensite dislocation density within the coating.

#### 3.1.2. Microstructure Evolution with Mn Content

**Figure 5:**
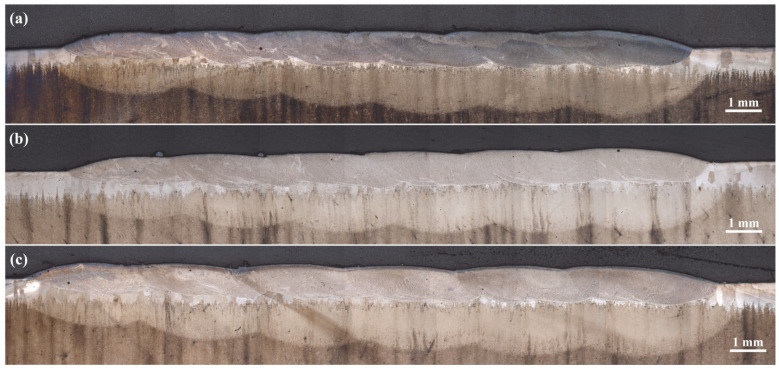
The single-layer macroscopic cross-sectional morphology of the Fe-based alloy coating with three different Mn contents: (**a**) 0 wt.%, (**b**) 1.0 wt.%, (**c**) 2.0 wt.%.

The single-layer macroscopic cross-sectional morphology of the Fe-based alloy coating with three different Mn contents is shown in Figure 5. The coating exhibits favorable forming quality and is free of defects such as cracks, and the Ra roughness value of the deposited coating is approximately 8–9 μm.

**Figure 6:**
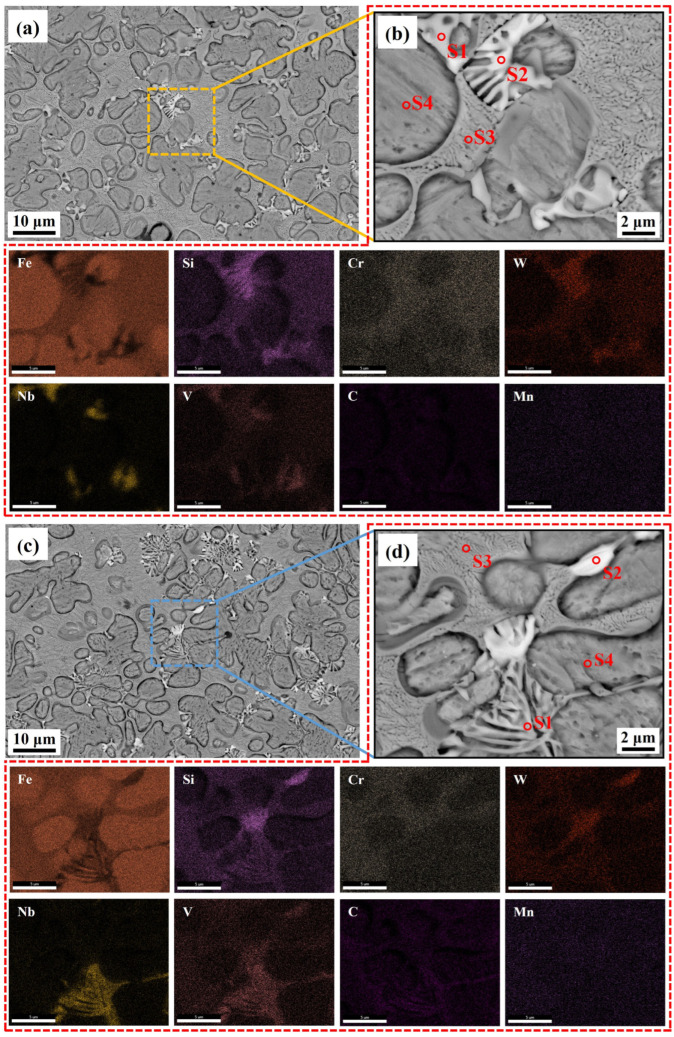

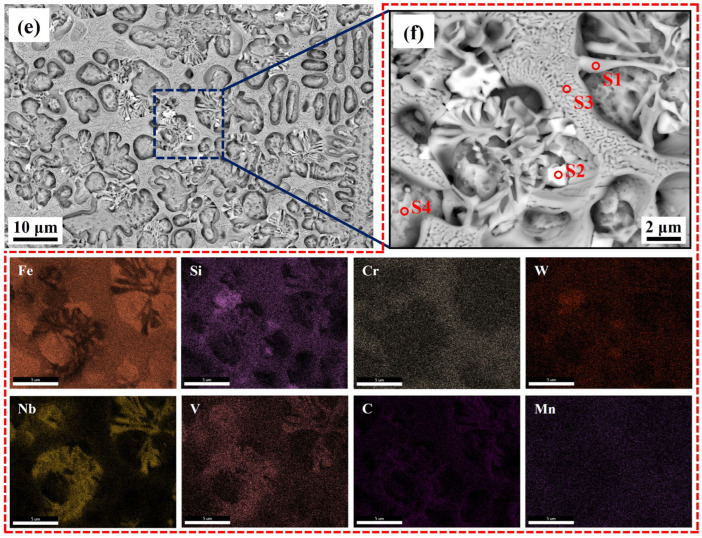
The microstructure of the Fe-based alloy coating with three different Mn contents, along with the corresponding EDS analysis maps: (**a**,**b**) 0 wt.%, (**c**,**d**) 1.0 wt.%, (**e**,**f**) 2.0 wt.%.

Figure 6 shows the microstructures of alloy powder coatings with three different Mn contents, prepared by laser cladding, in backscattered electron (BSE) mode, along with the corresponding EDS analysis maps. As shown in Figure 6a,b, three types of carbides with distinct morphologies, blocky, network, and irregular fishbone structures, are observed at the grain boundaries of the 0Mn alloy powder coating. To preliminarily identify the types of these three carbides, EDS area scans and point scans were performed on the regions and marked points corresponding to the three carbides precipitated at the grain boundaries. The results of the EDS area scans and point scans are shown in Figure 6 and Table 3, respectively. From the concentration maps of the major chemical elements (Fe, Si, Cr, W, Nb, V, C, and Mn), it is clearly evident that Nb and V are primarily enriched in the block-like carbides, while Si and W are mainly enriched in the irregular fishbone-like carbides. Additionally, Si and W are present in small amounts in the network-like carbides. Furthermore, in addition to the enrichment of Si and W, the network-structured carbides also exhibit enrichment of Cr. Furthermore, based on the combined results of point scanning and XRD analysis, it can be preliminarily inferred that the block-like carbides rich in Nb and V correspond to the MC phase, and the network-like carbides rich in Si, W, and Cr correspond to the M_2_C phase. Meanwhile, according to related studies [30], M_2_C eutectic carbide is a metastable phase, and decomposition of M_2_C + γ-Fe → M_6_C + MC occurs during the laser cladding process to form finer M_6_C and MC type carbides. Therefore, it can be inferred that the irregular fishbone-like carbides rich in Si and W correspond to the M_6_C phase.

As shown in Figure 6c,d, with the addition of Mn, the Nb-rich and V-rich MC-type carbides gradually refine, transforming from a blocky structure to a fishbone-like structure; simultaneously, the Si-rich, W-rich, and Cr-rich M_2_C-type carbides also refine significantly. This is because the addition of Mn alters the interfacial energy at the liquid–solid interface during solidification of the melt pool, promoting uniform carbide nucleation and thereby refining the carbide size. Furthermore, more Si-rich and W-rich M_6_C-type carbides precipitate at grain boundaries, causing the structure to transition from an irregular fishbone-like structure to a blocky structure.

As shown in Figure 6e,f, as the amount of Mn added increases, the reticulated M_2_C-type carbides and fishbone-shaped MC-type carbides become finer, while the blocky M_6_C-type carbides also become significantly finer, exhibiting a fine granular structure. Furthermore, the content of all three types of carbides increases significantly, and their distribution becomes more uniform. This is because manganese influences segregation behavior during the solidification of the melt pool, reducing carbide segregation at grain boundaries and promoting a more uniform distribution within the matrix.

**Figure 7:**
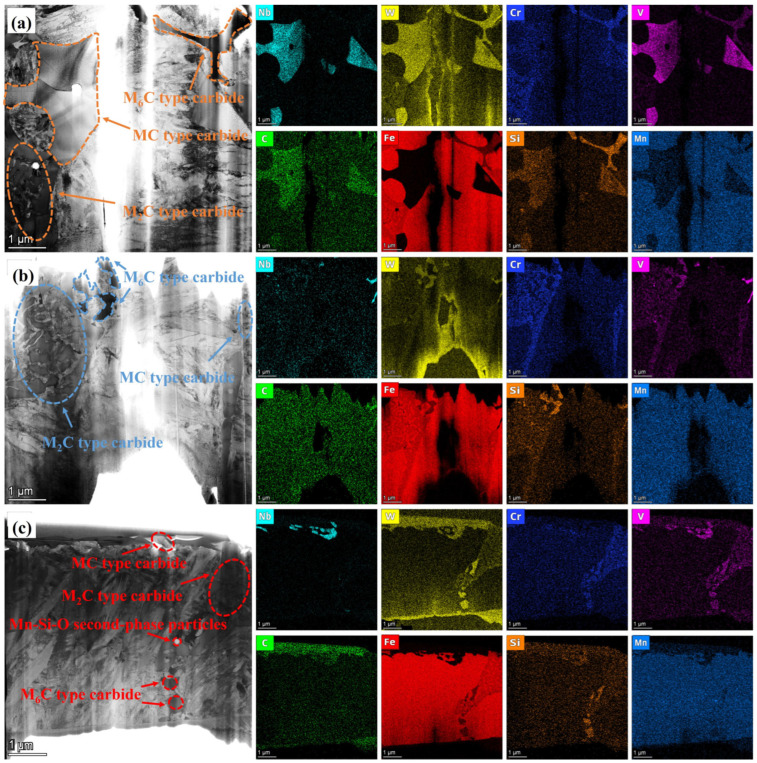
TEM bright-field images of the Fe-based alloy coating with three different Mn contents along with the corresponding mapping: (**a**) 0 wt.%, (**b**) 1.0 wt.%, (**c**) 2.0 wt.%.

Figure 7 shows TEM bright-field images and corresponding elemental distribution maps of iron-based alloy coatings with three different Mn contents. Figure 7a–c reveal the distinct presence of lath martensite in all three coatings, which contains high-density dislocations [31,32]. In order to reveal the microstructural details and determine the elemental enrichment states within each phase structure, STEM characterization was performed on the iron-based alloy coatings with three different Mn contents. The results confirmed that the elemental enrichment states were consistent with those observed in SEM. Meanwhile, the diffraction patterns of the three types of carbides precipitated at grain boundaries were identified, further confirming that the M_2_C, MC, and M_6_C carbides correspond to the W_2_C, NbC, and Fe_3_W_3_C phases, respectively.

**Figure 8:**
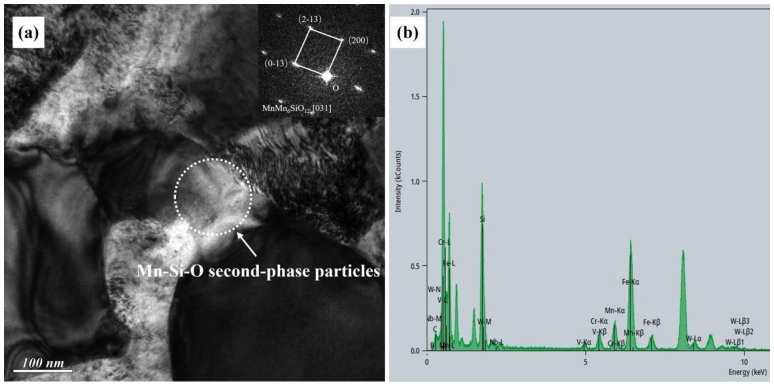
TEM bright-field images of the 2Mn coatings (**a**) and EDS analysis of Mn-Si-O second-phase particles (**b**).

In addition, the formation of Mn-Si-O second-phase particles was verified by TEM, as shown in Figure 7c; Mn-Si-O second-phase particles are suspended on the surface of M_2_C-type carbides. In order to further determine the types of Mn-Si-O second-phase particles in the 2Mn coating, the Mn-Si-O second-phase particles’ morphological features and crystal structures were analyzed using TEM, as shown in Figure 8. The Mn-Si-O second-phase particles shown in Figure 8a have a granular distribution and are SAED-labeled as a body-centered tetragonal crystal structure with a crystallographic band axis of [031].

### 3.2. Microhardness Analysis

**Figure 9:**
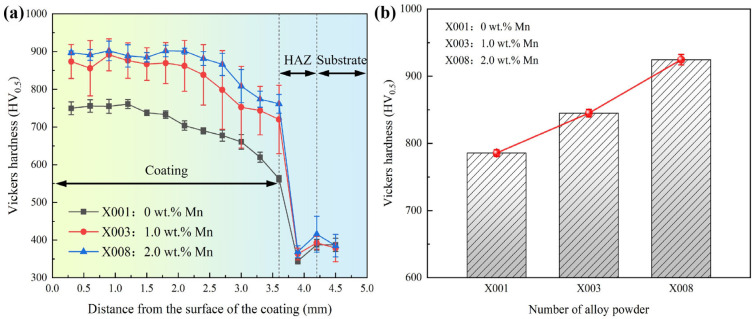
Analysis of the longitudinal microhardness distribution curves (**a**) and comparison of average microhardness data for the surface layer (**b**) of iron-based alloy coatings fabricated through laser cladding with three different Mn contents.

Figure 9a presents the cross-sectional microhardness depth profiles of the three iron-based alloy coatings with different Mn contents. The microhardness of all three coatings shows a general decreasing trend from the coating surface toward the substrate interface, which is associated with the distribution of carbides. In the laser-deposited coatings, carbides are mainly located in the upper and middle regions, leading to higher hardness near the top. All three coatings exhibit significantly higher microhardness than the substrate.

Figure 9b shows the average surface microhardness of the three alloy coatings. It is observed that the addition of Mn increases the microhardness. The average surface microhardness of the 1Mn coating (845 ± 6 HV_0.5_) is 7.51% higher than that of the 0Mn coating (786 ± 5 HV_0.5_). This improvement is attributed to the sporadic precipitation of Mn-Si-O second-phase particles, which effectively hinders dislocation motion. As the Mn content increases further, the microhardness is enhanced correspondingly. The 2Mn coating achieves the highest average surface microhardness of 925 ± 8 HV_0.5_. This further enhancement results from the continuous dissolution of Mn into the martensite lattice, causing lattice distortion that provides solid-solution strengthening and increases the resistance to dislocation movement, thereby markedly improving the microhardness of the coating [33].

### 3.3. Wear Behavior Analysis

**Figure 10:**
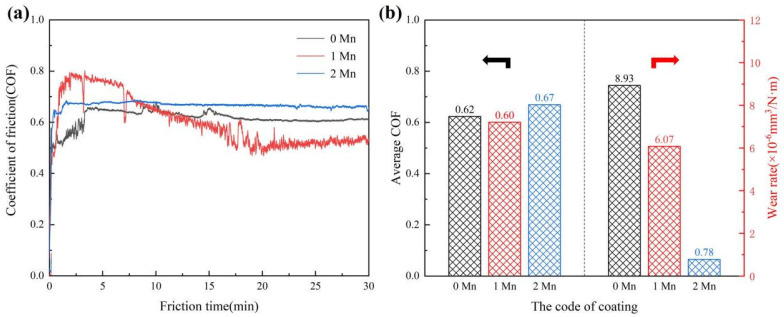
Coefficient of friction versus time curves (**a**), and comparison of wear rate data (**b**) of the Fe-based alloy coatings with different Mn contents.

Figure 10a shows the time-dependent friction coefficient curves of the three coatings with different Mn contents. A running-in phase is observed at the beginning of the friction and wear test. During this stage, the counterbody ball continuously presses into the coating surface, increasing the real contact area and causing surface damage, which leads to a rapid rise in the friction coefficient [34]. Compared with the other two coatings, the 1Mn coating exhibits a significantly longer running-in stage, indicating that its surface is less prone to plastic deformation and that a stable tribolayer forms more slowly [29].

To enable a more intuitive and reliable comparison, the average friction coefficients were calculated from the steady-state portion of the curves, as shown in Figure 10b. The average friction coefficients of the three coatings are roughly similar at 0.62, 0.60, and 0.67, respectively. Additionally, with increasing Mn content, the wear rate also decreases gradually. The addition of Mn introduces high-density dislocations in the coating. During plastic deformation, these dislocations become entangled and impede the motion of subsequently generated dislocations, thereby requiring higher stresses to induce further plastic deformation in the friction process. Moreover, the high-dislocation-density martensitic matrix enhances the work-hardening capacity of the coating, facilitating the rapid formation of an extremely hard surface layer that delays fatigue spalling. Additionally, Mn promotes the formation of a dense, well-adhered spinel-type oxide film during sliding, which reduces the friction coefficient and alleviates adhesive wear [35,36,37]. The synergistic effect of these three factors leads to a continuous decrease in the wear rate as the Mn content increases moderately. Specifically, the 0Mn coating exhibits the highest wear rate of 8.93 × 10^−6^ mm^3^/N·m, the 2Mn coating reaches the lowest value of 0.78 × 10^−6^ mm^3^/N·m, and the 1Mn coating shows an intermediate wear rate of 6.07 × 10^−6^ mm^3^/N·m. These results demonstrate that incorporating an appropriate amount of Mn into the coating material significantly improves the friction and wear performance, which is also consistent with the general trend that coating microhardness scales positively with wear resistance [38,39].

**Figure 11:**
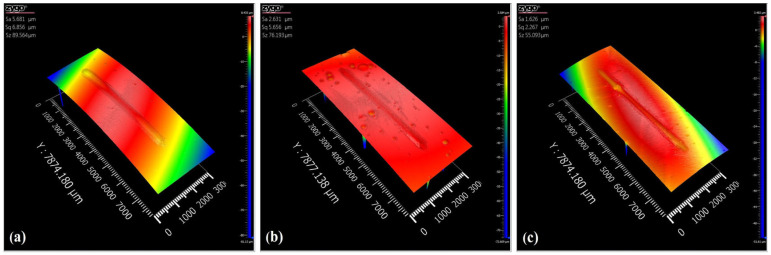
3D profile of wear tracks of the Fe-based alloy coatings with three different Mn contents: (**a**) 0 wt.%, (**b**) 1.0 wt.%, (**c**) 2.0 wt.%.

**Figure 12:**
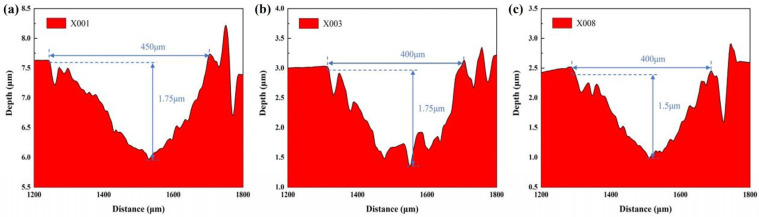
2D profile of wear tracks of the Fe-based alloy coatings with three different Mn contents: (**a**) 0 wt.%, (**b**) 1.0 wt.%, (**c**) 2.0 wt.%.

Figure 11 and Figure 12 display the 2D and 3D profile curves of the wear tracks, respectively. When no Mn is added, the wear track on the coating surface reaches its maximum width of 450 μm and maximum depth of 1.75 μm. As the Mn content increases, both the depth and width of the wear tracks decrease continuously, consistent with the trend in the wear rate. When the Mn content is 2.0 wt%, the wear track width and depth are reduced to 400 μm and 1.5 μm, respectively.

**Figure 13:**
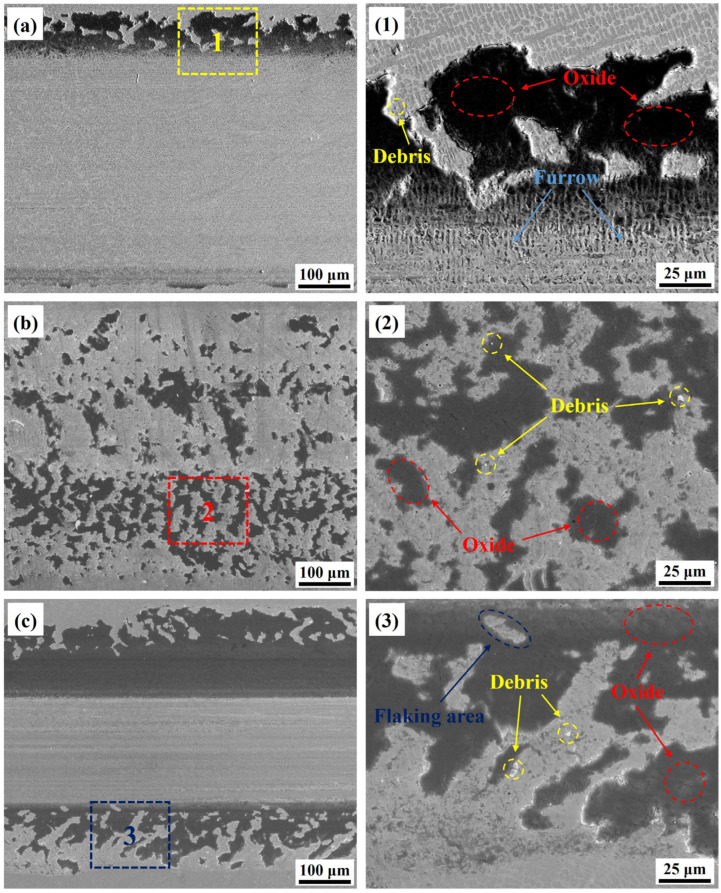
Surface morphology of abrasion marks of the Fe-based alloy coatings with three different Mn contents: (**a**) 0 wt.%, (**b**) 1.0 wt.%, (**c**) 2.0 wt.%.

Figure 13 shows the surface topographies of the wear tracks and the corresponding magnified views. The surface of the 1Mn coating is completely covered by a dense oxide layer, with only a small amount of wear debris visible at the edges. In contrast, the oxide layers of the 0Mn and 2Mn coatings are concentrated on both sides of the wear tracks. The oxide layers in the central region have spalled over a large area and been replaced by plowing grooves. Compared with the 0Mn coating, however, the 2Mn coating retains a noticeably larger oxide-covered area on both sides of the wear track, and a wide, flat, and smooth oxide layer is present on the upper side of the track. This indicates that the oxide layer on the 2Mn coating remained intact during the wear process without spalling or cracking, which helps to reduce wear and reasonably explains the significant decrease in the wear rate of the 2Mn coating. Based on the wear track morphologies of the three coatings, it can be inferred that the dominant wear mechanisms of all three are oxidative wear and abrasive wear [40].

**Figure 14:**
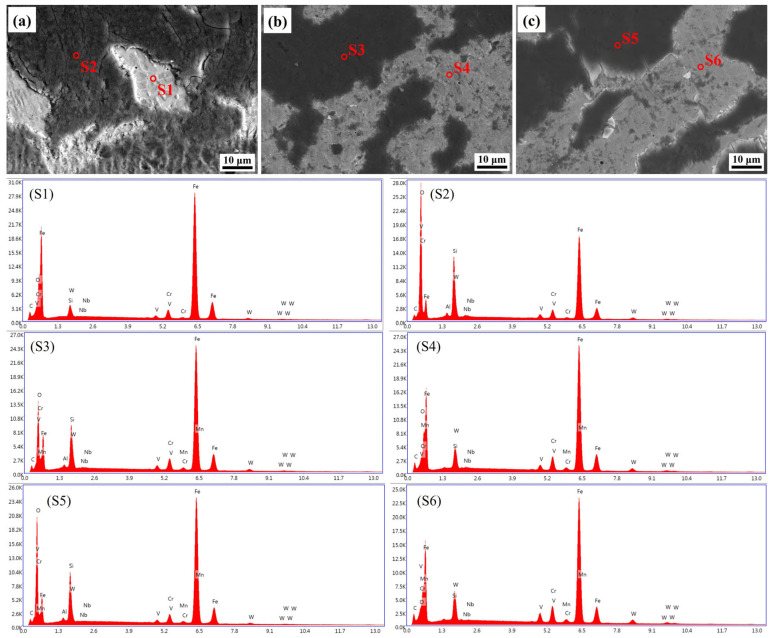
Selected point in images for EDS spectral analysis of the Fe-based alloy coatings wear tracks with three different Mn contents: (**a**) 0 wt.%, (**b**) 1.0 wt.%, (**c**) 2.0 wt.%, and the energy spectral analysis spectra of the labeled points 1, 2, 3, 4, 5 and 6 (**S1**–**S6**), respectively.

To further analyze the elemental composition of the micro-areas on the wear scar surfaces, six marked points were selected on the wear scar of each coating for EDS elemental analysis. The selected points and the corresponding test results are shown in Figure 14 and Table 4, respectively. The Si content in the oxide layer on the wear scar surface is significantly higher than the nominal Si content of the alloy powders and coatings (0.8~1.2 wt.%). This is attributed to the fracture of the Si_3_N_4_ ceramic balls during the wear test, which causes material transfer onto the wear scar. The transferred material reacts with oxygen in the air, forming new oxides on the wear scar surface. Under the high temperatures generated by friction, the transferred material reacts with oxygen in the air, forming new oxides on the wear scar surface [41].

## 4. Conclusions

This study is based on a novel Fe-C-B-X multicomponent iron-based alloy powder developed in previous research. By conducting comparative experiments to adjust the Mn content in the alloy powder, three iron-based alloy coatings with different Mn contents were successfully fabricated on the surface of 38CrMoAl steel using laser cladding technology. The study systematically investigated the influence of Mn content on the microstructural evolution and mechanical properties of high-strength, hard iron-based coatings produced via laser cladding. The specific conclusions are as follows:

(1) Changes in Mn content alter the morphology and size of carbides in the coating. In alloy powder coatings without added Mn, the carbides consist primarily of reticulated M_2_C-type, blocky MC-type, and fishbone-shaped M_6_C-type carbides. As the Mn content increases, the MC-type carbides gradually transform from a blocky to a fishbone-shaped structure. The fishbone-shaped M_6_C carbides first transform into block-shaped forms, ultimately resulting in a fine granular structure, and the sizes of all three types of carbides are refined.
(2) The three types of iron-based alloy powder coatings consist primarily of a martensitic matrix, retained austenite, and various M_x_C-type eutectic carbides (such as MC, M_2_C, M_6_C, etc.). Upon adding Mn to the coating material, scattered particles of a second phase (Mn-Si-O) precipitated within the coating. Simultaneously, the dislocation density in the martensite phase significantly increases, leading to a rise in the coating’s microhardness. When the mass fraction of Mn is 2.0 wt.%, the surface microhardness of the coating specimen reaches a maximum value of 925 ± 8 HV_0.5_.
(3) The wear mechanisms of the three iron-based alloy powder coatings all manifest as oxidative wear and abrasive wear. The addition of an appropriate amount of Mn to the coating material significantly improves the coating’s friction and wear properties. The average coefficients of friction for the three alloy powder coatings were roughly the same; however, when the mass fraction of Mn was 2.0 wt.%, the wear rate of the coated specimens reached a minimum of 0.78 × 10^−6^ mm^3^/N·m, a significant reduction compared to the 0Mn alloy powder coating.
(4) The addition of an appropriate amount of Mn to the coating material significantly improves the coating’s performance. However, a higher amount of Mn causes excessive carbide precipitation in the coating, leading to coarsening of the carbide grains and a loss of congruence with the substrate. Meanwhile, it severely weakens intergranular bonding, increases the coating’s susceptibility to cracking, and reduces its overall performance.

## Figures and Tables

**Table 1:** The main chemical composition of the substrate and three powders (wt.%).

Materials	C	Si	Mn	Cr	V	W	B	Nb	Mo	Ni	Al	Fe
38CrMoAl	0.39	0.20	0.44	1.48	-	-	-	-	0.18	0.10	1.03	Bal.
0Mn	1.0	1.0	-	4.0	1.7	8.0	0.9	1.5	-	-	-	Bal.
1Mn	1.0	1.0	1.0	4.0	1.7	8.0	0.9	1.5	-	-	-	Bal.
2Mn	1.0	1.0	2.0	4.0	1.7	8.0	0.9	1.5	-	-	-	Bal.

**Table 2:** Laser cladding process parameters.

Laser Power (W)	Scanning Speed (mm/s)	Powder Feeding Rate (g/min)	Protective Gas Flow Rate (L/min)	Preheating Temperature (°C)	Overlap Rate (%)
2200	16	10	15	200	50

**Table 3:** EDS chemical content of points on the surface of the Fe-based alloy coatings with three different Mn contents (wt.%).

Materials	Spot	Precipitated Phase	C	Si	Nb	V	Cr	Mn	Fe	W
0Mn	Spot 1	MC	9.30	0.94	41.74	13.18	1.83	-	15.59	17.42
	Spot 2	M_6_C	4.28	1.30	1.13	6.42	7.34	-	42.99	36.55
	Spot 3	M_2_C	2.40	0.26	0.31	2.98	6.26	-	74.61	12.48
	Spot 4	Matrix	2.68	0.89	0.02	0.93	2.73	-	88.93	3.82
1Mn	Spot 1	MC	10.34	1.09	20.78	8.18	2.82	0.51	44.20	12.08
	Spot 2	M_6_C	3.97	1.18	0.80	5.75	7.04	0.68	43.36	37.22
	Spot 3	M_2_C	1.52	0.19	0.29	2.97	6.95	0.97	73.41	13.70
	Spot 4	Matrix	2.42	0.83	0.06	0.99	2.88	0.55	88.79	3.48
2Mn	Spot 1	MC	9.91	0.48	24.41	8.12	3.61	0.85	38.37	14.25
	Spot 2	M_6_C	4.03	0.45	4.89	5.75	6.32	1.38	45.75	31.43
	Spot 3	M_2_C	4.48	0.32	1.82	2.92	6.49	1.50	68.63	13.83
	Spot 4	Matrix	2.91	1.01	0.44	1.35	3.20	1.24	85.36	4.49

**Table 4:** EDS chemical content of points on the wear scar surface of the Fe-based alloy coatings with three different Mn contents (wt.%).

Materials	Spot	C	O	Si	Nb	V	Cr	Mn	Fe	W
0Mn	S1	1.88	0.74	0.85	0.04	1.05	3.19	-	88.58	3.67
	S2	1.12	18.80	7.61	0.47	1.46	3.37	-	61.07	6.09
1Mn	S3	0.84	7.56	4.55	0.15	1.29	3.59	0.57	76.31	5.15
	S4	1.74	1.14	0.70	0.09	1.90	4.78	0.90	79.58	9.17
2Mn	S5	0.75	11.37	5.39	0.02	1.24	2.99	0.80	75.97	1.48
	S6	2.56	0.01	0.50	-	3.13	5.50	0.68	76.61	11.01

## Data Availability

The raw data supporting the conclusions of this article will be made available by the authors on request.
